# Review: Serum Biomarkers of Lung Fibrosis in Interstitial Pneumonia with Autoimmune Features—What Do We Already Know?

**DOI:** 10.3390/jcm11010079

**Published:** 2021-12-24

**Authors:** Ewa Miądlikowska, Patrycja Rzepka-Wrona, Joanna Miłkowska-Dymanowska, Adam Jerzy Białas, Wojciech Jerzy Piotrowski

**Affiliations:** 1 Department of Pneumology, Medical University of Lodz, 90-153 Łódź, Poland; ewa.miadlikowska@umed.lodz.pl (E.M.); joanna.milkowska-dymanowska@umed.lodz.pl (J.M.-D.); 2 Department of Pneumonology, School of Medicine in Katowice, Medical University of Silesia, 40-055 Katowice, Poland; patrycja.rzepka2@gmail.com; 3 Department of Pathobiology of Respiratory Diseases, Medical University of Lodz, 90-153 Łódź, Poland; adam.bialas@umed.lodz.pl

**Keywords:** interstitial pneumonia with autoimmune features, IPAF, pulmonary fibrosis, biomarkers, KL-6, SP-A, SP-D, circulating fibrocytes, CXCL

## Abstract

Interstitial pneumonia with autoimmune features (IPAF) belongs to a group of diseases called interstitial lung diseases (ILDs), which are disorders of a varied prognosis and course. Finding sufficiently specific and sensitive biomarkers would enable the progression to be predicted, the natural history to be monitored and patients to be stratified according to their treatment. To assess the significance of pulmonary fibrosis biomarkers studied thus far, we searched the PubMed, Medline and Cochrane Library databases for papers published between January 2015 and June 2021. We focused on circulating biomarkers. A primary review of the databases identified 38 articles of potential interest. Overall, seven articles fulfilled the inclusion criteria. This review aims to assess the diagnostic and prognostic value of molecules such as KL-6, SP-A, SP-D, circulating fibrocytes, CCL2, CXCL13, CXCL9, CXCL10 and CXCL11. All of these biomarkers have previously been studied in idiopathic pulmonary fibrosis (IPF) and connective tissue disease-associated interstitial lung disease (CTD-ILD). IPAF is a disorder of a heterogeneous nature. It explains the lack of coherent observations in terms of correlations with functional parameters. There is still no meta-analysis of pulmonary fibrosis biomarkers in IPAF. This is mainly due to the heterogeneity of the methodology and groups analysed in the research. More research in this area is needed.

## 1. Introduction

Interstitial pneumonia with autoimmune features (IPAF) is a relatively novel disorder developed in 2015 by the European Respiratory Society/American Thoracic Society Task Force on Undifferentiated Forms of Connective Tissue Disease-Associated Interstitial Lung Disease [1]. This document sparked scientific interest in IPAF and multiple, mainly retrospective, studies on IPAF cohorts. The mentioned publication aimed to identify, describe and study patients with interstitial lung disease (ILD) who display some symptoms of autoimmunity, but do not meet established criteria for any connective tissue disease.

It is estimated that approximately 7% of ILD patients may be diagnosed with IPAF [2]. It affects mostly women in the 6–7th decades of their lives. The most commonly reported extrapulmonary symptoms are Raynaud’s phenomenon, arthritis, morning stiffness and “mechanic’s hands”. Often, patients may also present with a dry cough, shortness of breath and fatigue. Anti-nuclear antibodies (ANA) were the most frequently identified antibodies in blood serum serological tests in patients with IPAF. The predominant pattern in high-resolution computed tomography (HRCT) was nonspecific interstitial pneumonia (NSIP), which is also characteristic of lesions accompanying most systemic connective tissue diseases [3,4,5,6,7,8]. Distinct results were described in Oldham’s research—the cohort study reports a high proportion of usual interstitial pneumonia (UIP). This is mostly likely due to the fact that the study was conducted retrospectively in the reference centre for IPF patients.

Previous studies suggest that only a small fraction of patients with IPAF can be diagnosed with a specific systemic connective tissue disease over time [6]. The prognosis in patients with IPAF seems to be better, according to some authors, than in those with idiopathic pulmonary fibrosis (IPF). Moreover, it appears that the course of the disease may differ depending on the HRCT-based pattern: patients with the NSIP pattern had a longer survival time than those with the UIP-pattern [3]. Patients with IPAF appear to have fewer exacerbations than other patients with IIP [9].

Prospective studies in multidisciplinary and multicentre settings provide information about best clinical practices for the diagnosis, treatment and management of the cohort. Currently, patients fulfilling IPAF diagnostic criteria are taking part in treatment trials with pirfenidone [10]. Most likely, the conclusions drawn from the trials will result in the further improvement and specification of the 2015 criteria [11,12].

## 2. Biomarkers

The term “biomarker” can be defined as “a specific characteristic that is measured as an indicator of normal biologic processes, pathogenic processes, or responses to an exposure or intervention, including therapeutic interventions” [13]. A broader definition of pulmonary fibrosis biomarkers may include the results of respiratory function tests, imaging or biochemical molecules that are detectable in blood, bronchoalveolar lavage or lung tissue. Biomarkers could be used for a variety of purposes: diagnostic, prognostic, therapeutic or to identify patients with a predisposition to developing a certain disease.

IPAF belongs to a group of diseases called interstitial lung diseases (ILDs), which are disorders of a varied prognosis and course. They are characterised by the destruction of lung tissue by inflammation and fibrosis. The pathogenesis of pulmonary fibrosis is not fully understood. It is known to be caused by immune system activation, diffuse remodelling of the lung parenchyma, the presence of excess extracellular matrix or irreversible scarring [14,15].

Four main groups of circulating ILD candidate biomarkers, categorised by the pathophysiology pathways, can be distinguished as follows [16,17]:

- alveolar epithelial cell damage and dysfunction (KL-6, SP-A, SP-D);
- aberrant fibrogenesis and matrix remodelling (MMP7, MMP3, LOXL2, HSP47, IGFBPs, periostin, circulating fibrocytes, fibrillin-1, osteopontin);
- damaged endothelium (IL-8, ET-1, VEGF);
- immune dysregulation and inflammation (CCL18, YKL-40, ICAM, VCAM, E-selectin, IL-6, CXCL-13, anti-HSP70 IgG, BLyS, serum RAGE).

A growing body of evidence suggests their role in pulmonary fibrosis in patients with idiopathic pulmonary fibrosis (IPF), which is the most extensively studied fibrotic interstitial lung disease, but also in patients with CTD-ILD.

## 3. Materials and Methods

We searched the PubMed, Medline and Cochrane Library databases for papers published between January 2015 and June 2021 using the following combination of terms: (“interstitial pneumonia with autoimmune features” OR IPAF) AND (biomarker OR biomarkers OR molecule). Abstracts and articles not written in English, trials, reviews and letters were excluded.

## 4. Results

**Figure 1:**
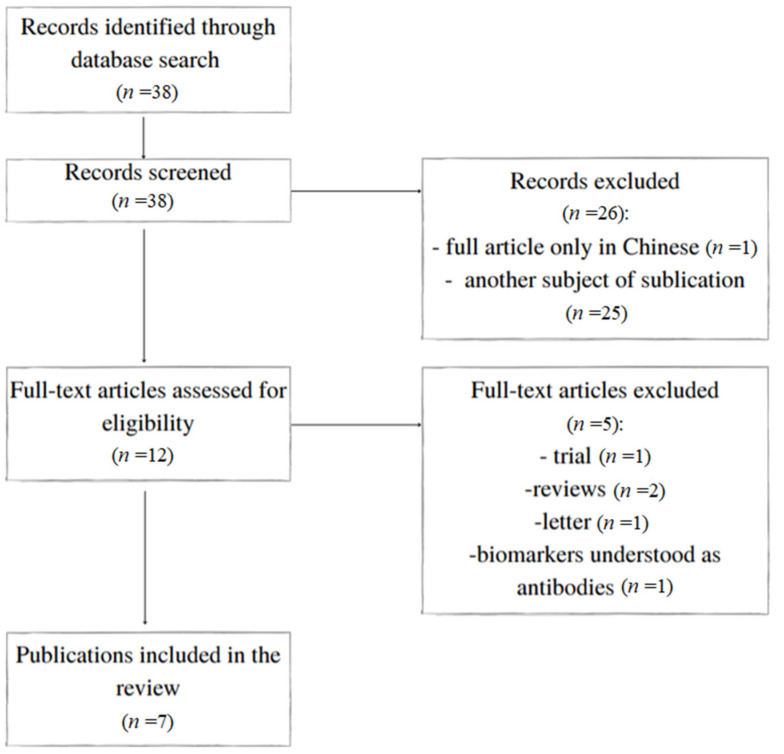
Flowchart of the study.

A primary review of the databases identified 38 articles of potential interest. Twenty-six were excluded based on their title or abstract, resulting in 12 references being examined for the full text (Figure 1). Overall, seven articles fulfilled the inclusion criteria. They are presented in Table 1. The biomarkers and their validity are summarised in Table 2.

### 4.1. KL-6

Krebs von den Lungen-6 (KL-6), a high molecular weight glycoprotein, also known as human mucin-1 (MUC1), is mainly produced by damaged or regenerating alveolar type II pneumocytes. It can also be found on the epithelial cells of the stomach, pancreas and oesophagus. The glycoprotein, described for the first time by Kohno et al., plays an important role in the morphogenesis and development of foetal lungs and exhibits chemotactic properties for fibroblasts [24,25].

KL-6 levels were significantly higher in the patients with IPAF than in the patients with non-IPF interstitial fibrosis, non-fibrotic lung diseases, pneumonia and a healthy group [18,19,20,22]. The biomarker level, when compared to IPF, was varied depending on the study: it was significantly higher in IPAF in Kameda’s study, but comparable in Xue’s study [18,19].

Moreover, in three studies, the serum KL-6 levels showed a negative correlation with the transfer factor for carbon monoxide (T_LCO_) [19,20,22]. The results of the studies regarding the correlation between KL-6 and the percentage of predicted forced vital capacity value (%FVC) differed from study to study: there was no significant correlation with %FVC and the percentage of predicted forced expiratory volume in one second value (%FEV_1_) in Wang’s publication, whilst the association with %FVC was described in Xue’s article [20,22].

A significant positive correlation with the severity of interstitial lung lesions in the IIP group (including IPAF, although the disease was not separately analysed) was also observed [19].

Furthermore, Wang proved that the post-treatment KL-6 serum levels were significantly increased compared to the pre-treatment ones in patients with progressive disease. The opposite effect was noted in the improvement group. The results suggest that KL-6 may be used as a biomarker to monitor the progression of pulmonary fibrosis in patients with IPAF [20]. However, the results were not fully confirmed by Yamakawa’s study [21].

In Xue’s prospective study with a 52-week follow-up, there was a positive correlation between the KL-6 serum levels and CT scores in the aggravation group. The investigators did not observe any correlation in the improvement or stable groups. Furthermore, there was no significant correlation between KL-6 and autoimmune factors [22].

To sum up, the KL-6 level seems to be higher in IPAF than in a healthy group and non-fibrotic lung diseases. There is a negative correlation between the serum level of this molecule and T_LCO_. In the aggravation groups, the KL-6 levels correlate with the degree of lung involvement.

### 4.2. SP-A and SP-D

Surfactant proteins SP-A and SP-D are large hydrophilic proteins—collagen-containing C-type lectins called collectins. They are produced by Clara cells and type II alveolar epithelial cells. SP-A and SP-D are important for innate immune mechanisms and help to resolve inflammation on the alveolar surface [26,27,28]. They are among the most thoroughly studied biomarkers in IPAF.

The SP-A and SP-D levels were higher in the IPAF patients than in a healthy group, the patients with pneumonia or non-fibrotic lung diseases [19,20,22]. Furthermore, the SP-D serum levels were lower in the IIP non-IPF group than in the IPAF patients [19]. The SP-A level cannot be used to distinguish between IPAF and CTD-ILD patients [22].

In Xue’s publication, a negative correlation of SP-A serum levels and T_LCO_ was observed in the IIP group (including 27/69 patients with IPAF, although this group was not separately investigated). A negative correlation was also noted with FEV_1_ and FVC pulmonary ventilatory function parameters in that group [19]. The observation was partially confirmed by Wang’s study: the investigators proved a negative correlation between SP-A serum levels and changes in T_LCO_, FEV_1_ and FVC (delta T_LCO_, delta FEV_1_, delta FVC) results after treatment. However, there was no significant correlation between the serum SP-A levels and %FVC or %FEV_1_ in the said article and Xue’s prospective study [20,22].

Moreover, in his article, Wang described a suspected prognostic role of SP-A: the pre-treatment biomarker levels were significantly lower than the post-treatment ones in patients with the progressive type of IPAF. Additionally, a significant positive correlation was found between changes in the KL-6 and SP-A levels [20]. Unfortunately, the prognostic role was not confirmed in the case of the SP-D serum level in the other studies: the biomarker slope was not significantly different between disease courses.

In Xue’s prospective study, a significant difference was noted in the SP-A serum levels at baseline and 52 weeks. In the aggravation group, the biomarker also correlated with HRCT scores. In contrast, the correlation was not found in the improvement and stable groups. Additionally, no relationship was observed between the SP-A serum levels and autoantibodies [22].

In conclusion, it can be said that there is a negative correlation between the level of SP-A and the results of respiratory function tests (T_LCO_, FEV_1_, FVC) in patients with IPAF. In the progressive group, the level of this molecule increases over time. Moreover, in IPAF, the SP-A and SP-D levels were higher than in the patients with pneumonia, non-fibrotic lung diseases and a healthy group.

### 4.3. Circulating Fibrocytes

Circulating fibrocytes are cells derived from bone marrow. They have the features of hematopoietic and mesenchymal cells. The cells are involved in inflammatory reactions, including autoimmune ones, as well as fibrosis and wound healing [29].

There is one study in which scientists examined the concentration of circulating fibrocytes in patients with autoimmune interstitial lung diseases (including IPAF). Unfortunately, the IPAF group was not separately analysed; hence, it is impossible to draw any conclusions. Interestingly, the concentrations of circulating fibrocytes were higher in the patients with autoimmune interstitial lung disease than in the control group. The biomarker serum levels declined with the use of immunosuppressive therapy [16].

### 4.4. CCL2

Chemokine ligand 2—monocyte chemoattractant protein-1 (MCP-1)—is another profibrotic chemokine associated with pulmonary fibrosis. CCL2 is expressed in macrophages, alveolar epithelial cells and lung vascular endothelium in pulmonary fibrosis [19,30,31].

The CCL2 serum levels showed a negative correlation with T_LCO_ in the IIP group including IPAF, although IPAF patients were not distinguished. The CCL2 levels were notably higher in the IPAF group than in a healthy one [19].

### 4.5. CXCL13

Similar to CCL2, in Xue’s study, the serum levels of CXCL13 were significantly lower in the patients with pneumonia and the normal controls than in the IIP group (including IPAF patients). Their negative correlation with T_LCO_ was also noted. Additionally, the CXCL13 serum levels were higher in the IPAF group than in the IPF group [19].

### 4.6. CXCL9, CXCL10, CXCL11

CXCL9 (C-X-C motif chemokine), CXCL10 and CXCL11 are cytokines responsible for the recruitment of immune cells at inflammation sites. They also have an impact on angiogenesis [32].

Kameda’s study showed that the serum levels of the biomarkers in the IPAF patients were significantly elevated compared to the IPF patients. CXCL9, CXCL10 and CXCL11 serum levels correlated with %FVC, C-reactive protein and alveolar-arterial oxygen difference. Furthermore, the CXCL9 and CXCL10 serum levels also correlated with the bronchoalveolar lavage fluid (BALF) levels.

It is worth noting that a positive correlation was observed between the CXCL9 and CXCL11 pre-treatment serum levels and the annual changes in FVC in the patients with IPAF treated with immunosuppressive drugs. This observation provides the basis for further studies of the prognostic significance of these biomarkers [18].

### 4.7. Other Biomarkers

Kameda reported that the TNF-alpha levels in IPAF patients were higher than in the IPF group and lower than in patients with collagen vascular diseases–associated interstitial lung disease (CVD-ILD), however, without statistical significance [18].

In Liang’s study, the investigators noted that the CXCL1, IL-4, IL-13, IL-6 and IL-17 serum levels were higher in the patients with IPAF than in those with other types of IIP, COPD and healthy individuals. Furthermore, the CXCL1 levels in the acute exacerbation phase were notably higher than in the stable phase. The biomarkers were also negatively correlated with T_LCO_ [23].

## 5. Discussion

The role of biomarkers in ILDs’ diagnosis, treatment choice or prognostication thinking about patients’ prognosis has still not been established. There is little research specifically on patients with IPAF, thus the selection of particular molecules was based on studies in other ILD patients.

The biomarker that appears most frequently in the cited studies is KL-6, which is not without a reason: KL-6 is one of the most thoroughly investigated molecules in ILDs. The level of the biomarker increases in damaged alveolar tissue affected by interstitial pneumonia and the biomarker subsequently enters the circulation [33].

According to the articles included in the review, serum KL-6 may differentiate various clinical entities. In Kameda’s study, it was much higher in IPAF than in IPF, in Xue’s study, the levels were comparable, whereas Yamakawa suggests higher KL-6 levels in IPAF than in non-IPAF NSIP [18,19,21].

Its level is elevated in various ILDs (IPF, CTD-ILD, HP) without significant differences between these diseases [34,35,36,37]. Predictive mortality and survival values in IPF patients were suggested [38]. Furthermore, high KL-6 levels were associated with pulmonary function disruption (%FVC, %T_LCO_) in CTD-ILD patients and led to poor prognosis [39,40].

A negative correlation between KL-6 and %FVC was noted, although not in all the IPAF studies, while a correlation with T_LCO_ was revealed in all of them. A similar relationship was observed in Sokai’s study in the IPF group [41]. It is suggested that T_LCO_ may be a better biomarker for progression monitoring. That may be due to the fact that, in many patients, emphysema or pulmonary hypertension coexists with pulmonary fibrosis, resulting in a reduction in T_LCO_ with preserved FVC.

Contrary to other studies, the situation may be different in antisynthetase syndrome. KL-6 levels may be extremely elevated in patients with inflammatory myositis-associated subacute ILD, regardless of the disease severity [42]. High concentrations of KL-6 may result from completely different pathological processes, the marker is non-specific and in a disease entity such as ASS, where the inflammatory component is dominant, it may not be of prognostic significance. That is worth mentioning because patients with oligosymptomatic antisynthetase syndrome (ASS) can be distinguished among IPAF patients. The ASS spectrum is heterogeneous and three different diagnostic criteria—EULAR/ACR, Connor’s and Solomon’s criteria—were proposed over the last few years. Depending on the doctor’s decision, patients who do not meet EULAR/ACR or Solomon’s criteria may be diagnosed with IPAF or ASS according to the broadest Connor’s criteria [43].

The next extensively investigated molecules in ILDs are SP-A and SP-D, which are important markers of alveolar injury [41,44]. According to research, SP-A and SP-D levels were elevated in ILD, regardless of the type of disease. The levels of these molecules were comparable between IPAF and IPF patients. The molecules turned out to be strong predictors of mortality in IPF in three studies [26,27,28].

Among CTD-ILDs, patients with scleroderma are the most frequently studied group in terms of pulmonary fibrosis biomarkers. In the studies, SP-A and SP-D serum levels were negatively correlated with pulmonary function tests [40,45]. In Takahashi’s research, a correlation between SP-D and the extent of ground-glass change on HRCT was found, which was not confirmed in further studies [46,47,48]. The fact is interesting in the context of Xue’s observation: he suggested that SP-A serum levels correlated with CT scores in IPAF patients [22].

The authors became interested in another biomarker, i.e., circulating fibrocytes, as their elevated levels were associated with worse survival and negatively correlated with T_LCO_ and FVC in IPF [49]. Moreover, increased biomarker values were observed in autoimmune diseases, such as systemic scleroderma, rheumatoid arthritis or Graves’ disease, which suggests a possible role of fibrocytes in autoimmunity [29]. That is why the biomarker may be important in IPAF patients and requires further studies.

The next molecule investigated in IPAF was CCL2. Previous studies showed that it plays a role in inflammation and innate immunity. A profibrotic effect in systemic sclerosis (SSc) and rheumatoid arthritis (RA) was suggested [50,51,52]. Interestingly, although CCL2 serum concentrations were elevated in IPF patients, Raghu’s trial on the use of carlumab, a CCL inhibitor, in these patients did not show any notable impact on pulmonary function tests [53].

One of the most interesting biomarkers described in our review is CXCL1. In Liang’s study, its level was significantly higher in IPAF compared to IIP. Its level was also highly associated with the severity of the disease. Such a correlation did not occur in IIP patients, which may mean that the CXCL1-CXCR2 axis is connected with the IPAF pathogenic mechanism [23].

Another molecule—CXCL13—was taken into consideration in the above publications due to the earlier studies of IPF patients. In Vuga’s study, the CXCL13 levels were several times higher in IPF patients compared with chronic obstructive pulmonary disease (COPD) and a healthy group. Moreover, the IPF patients with the highest concentration of this chemokine had a lower six-month survival rate. The CXCL13 levels were higher in patients with pulmonary hypertension exacerbations. This molecule is considered a marker of the advanced IPF disease [31,54].

The other chemokines—CXCL9, CXCL10, CXCL11—were interesting in the context of IPAF because of their suspected role in the inflammatory pathophysiology of ILD and CVDs [32,55,56,57]. These molecules were also studied in the context of sarcoidosis. The role of the chemokines CXCL9, CXCL10 and CXCL11 in the pathogenesis of chronic sarcoidosis and the correlation of their level with respiratory results are described [58,59,60]. In IPAF patients, the serum levels of these molecules are intermediate between IPF and CTD-ILD, which may reflect the level of autoimmune inflammation. Furthermore, patients with higher pre-treatment concentrations of CXCL9 and CXCL11 in both IPAF and CTD-ILD seem to respond better to immunosuppressive therapy [18].

It is very important to find biomarkers that predict the pulmonary fibrosis progression in IPAF. However, it is a disorder of a heterogeneous nature. This group of patients may include both patients with progressive fibrosis and patients with inflammatory patterns. This explains the lack of coherent observations in terms of correlations with functional parameters but offers great opportunities for prospective prognostic and predictive assessment in the future.

Antifibrotic drugs (pirfenidone and nintedanib) have been recently proposed as a therapeutic option for patients with pulmonary fibrosis other than IPF. Nintedanib was proven to be effective in progressive fibrosis ILD (PF-ILD) in INBUILD Trial [61]. Pirfenidone was studied in patients with unclassifiable lung fibrosis and was also shown to slow down the disease progression [10]. IPAF patients were included in both trials. Progressive phenotype occurs only in a proportion of IPAF patients [62]. Therefore, it is essential for the recruitment to antifibrotic treatment to prove the risk of pulmonary fibrosis progression. Biomarkers may help select patients who could benefit from such a treatment.

## 6. Conclusions

Only a few biomarkers have been tested in IPAF. In the analysed research, the KL-6, SP-A and SP-D levels were higher in IPAF than in a healthy group and non-fibrotic lung diseases. The serum levels of CXCL13, CXCL-9, CXCL10 and CXCL11 in IPAF patients were elevated compared to IPF patients. In the cited articles, a negative correlation was described between KL-6, CXCL-1, IL-4, IL-13, IL-6 and IL-17 serum levels and T_LCO_, between SP-A and the results of a respiratory function tests (T_LCO_, FEV_1_, FVC). The serum levels of CXCL9, CXCL10 and CXCL11 correlated with %FVC.

The knowledge of pulmonary fibrosis biomarkers is still insufficient, both in IPAF and other ILDs. Being a relatively new disease entity, IPAF provides a field for a lot of research. The molecules worth considering are, among others, matrix metalloproteinase-7 (MMP7), chemokine ligand 18 (CCL18) and YKL-40, which were biomarkers previously studied in IPF and CTD-ILD groups of patients.

Our review has many limitations. The reported studies are mainly retrospective and single centre. They were mostly conducted in Asia. Therefore, we do not have any data showing possible differences or similarities between different ethnic groups. However, available data in IPF confirm the usefulness of biomarkers in various ethnic groups. There is still no meta-analysis of pulmonary fibrosis biomarkers in IPAF. This is mainly due to the heterogeneity of the methodology and the groups analysed in the research. Furthermore, we have too little research to reliably compare the research on fibrotic biomarkers studied in patients with IPAF and other diseases (e.g., CTD-ILD, IIPs). More research in this area is needed. There is one ongoing project registered on the ClinicalTrials.gov website concerning the identification of IPAF diagnostic markers (ClinicalTrials.gov Identifier: NCT03870828).

Revealing the pathogenesis of IPAF is fundamental to a better understanding of the mechanisms leading to pulmonary fibrosis in the disorder. Finding sufficiently specific and sensitive biomarkers of pulmonary fibrosis in the condition would enable the progression to be predicted, the natural history to be monitored and patients to be stratified according to their treatment.

## Figures and Tables

**Table 1:** Studies included in the review.

Authors	Journal	Doi	Country	Year	Type of Study	Study Groups	Biomarkers
Kameda M et al. [18]	PLoS One	10.1371/journal.pone.0241719	Japan	2020	Single centre	102 participants: 35 IPAF, 51 IPF, 16 CVD-ILD (5 SSc, 4 RA, 2 PM, 2 microscopic polyangiitis, 2 mixed connective tissue disease, 1 Sjogren syndrome) Untreated at diagnosis	CXCL9, CXCL10, CXCL11, KL-6, SP-A, SP-D, CCL3, CCL7, CCL17, Fas-L, IL-6, IL-, IL-10, IL-18, TNF-alpha, TNFSF14
Xue M et al. [19]	Respiration	10.1159/000503689	China	2019	Single centre	69 patients with IIP: 19 IPF, 23 N-IPF, 27 IPAF Control groups: 20 age- and gender-matched patients with pneumonia, 15 uninfected individuals as controls	KL-6, SP-A, SP-D, CCL2, CCL13
Wang J et al. [20]	BMC Pulmonary Medicine	10.1186/s12890-020-01336-y	China	2020	Single centre	64 patients with IPAF (36 patients with follow-up > 3 months), 41 patients with non-fibrotic lung diseases	KL-6, SP-A, SP-D
Yamakawa H et al. [21]	Respiratory Investigation	10.1016/j.resinv.2019.03.006	Japan	2019	Single centre	75 patients with idiopathic fibrotic NSIP: 50 IPAF, 25 non-IPAF	KL-6, SP-D
Xue M et al. [22]	Medicine	10.1097/MD.0000000000024260	China	2021	Single centre	65 patients with IPAF control group: 30 age-matched healthy individuals (follow-up: 52 weeks)	KL-6, SP-A
Odackal J et al. [16]	ERJ Open Research	10.1183/23120541.00481-2020	USA	2020	Single centre	50 patients with autoimmune ILD: 18 IPAF, 5 RA, 3 MCTD, 13 myositis-related, 11 SSc. Control group: 26 healthy individuals	Circulating fibrocytes
Liang M et al. [23]	Scientific Reports	10.1038/srep38949	China	2016	Single centre	38 patients with IPAF, 81 patients with IIP, 36 patients with chronic obstructive pulmonary disease (COPD)	CXCL1

IPAF—interstitial pneumonia with autoimmune features, CVD-ILD—collagen vascular diseases–associated interstitial lung disease, SSc—systemic sclerosis, RA—rheumatoid arthritis, PM—polymyositis, IIP—idiopathic interstitial pneumonia, IPF—idiopathic pulmonary fibrosis, N-IPF—non-idiopathic pulmonary fibrosis, NSIP—nonspecific interstitial pneumonia, ILD—interstitial lung disease, MCTD—mixed connective tissue disease, CXCL9—chemokine ligand 9, CXCL10—chemokine ligand 10, CXCL11—chemokine ligand 11, KL-6—Krebs von den Lungen-6, SP-A—surfactant protein A, SP-D—surfactant protein D, CCL3—C-C motif chemokine ligand 3, CCL7—C-C motif chemokine ligand 7, CCL17—C-C motif chemokine ligand 17, FasL—Fas Ligand, IL-6—interleukin-6, IL-10—interleukin-10, IL-18—interleukin-18, TNF-alpha—tumour necrosis factor alpha, TNFSF14—TNF superfamily member 14, CCL2—C-C motif chemokine ligand 2, CCL13—C-C motif chemokine ligand 13, CXCL1—chemokine ligand 1.

**Table 2:** Circulating biomarkers associated with pulmonary fibrosis in IPAF.

Biomarker	Study	Country	Diagnosis	Severity	Prognosis
KL-6	Kameda M et al. [18] Xue M et al. [19,22] Wang J et al. [20] Yamakawa H et al. [21]	China, Japan	+	+	+
SP-A	Xue M et al. [19,22] Wang J et al. [20]	China	+	+	+
SP-D	Xue M et al. [19,22] Yamakawa H et al. [21]	China, Japan	+	+	-
CXCL13	Xue M et al. [19]	China	+	Not studied	Not studied
CXCL9, CXCL10, CXCL11	Kameda M et al. [18]	Japan	+	+	+
CXCL1	Liang M et al. [23]	China	+	+	+

‘+’ means that something is proved; KL-6—Krebs von den Lungen-6, SP-A—surfactant protein A, SP-D—surfactant protein D, CXCL13—chemokine ligand 13, CXCL9—chemokine ligand 9, CXCL10—chemokine ligand 10, CXCL11—chemokine ligand 11, CXCL1—chemokine ligand 1.
